# Salivary trefoil factor family peptide 3 (TFF3) and flow rate in persons with and without obstructive sleep apnea: A preliminary study

**DOI:** 10.1002/cre2.746

**Published:** 2023-05-14

**Authors:** Supanigar Ruangsri, Gintawat Doolgindachbaporn, Worrapon Chokwatwikul, Kanchanaporn Wattanawareekul, Subin Puasiri, Kittisak Sawanyawisuth

**Affiliations:** ^1^ Department of Oral Biomedical Science, Faculty of Dentistry Khon Kaen University Khon Kaen Thailand; ^2^ Neuroscience Research and Development Group (NRDG) Khon Kaen University Khon Kaen Thailand; ^3^ Institute of Dentistry Suranaree University of Technology Nakhon Ratchasima Thailand; ^4^ Department of Preventive Dentistry, Faculty of Dentistry Khon Kaen University Khon Kaen Thailand; ^5^ Department of Medicine, Faculty of Medicine Khon Kaen University Khon Kaen Thailand

**Keywords:** age, diagnosis, neck circumference

## Abstract

**Objectives:**

Obstructive sleep apnea (OSA) is one of the most common chronic diseases. Trefoil factor family 3 (TFF3) peptides are secreted by major and minor salivary glands and may be involved in the pathogenesis of OSA. This study aimed to evaluate salivary TFF3 and flow rate between those with and without OSA.

**Material and methods:**

This was a prospective experimental study that enrolled patients with OSA and non‐OSA. Total unstimulated saliva was collected, the salivary flow rate was measured, and the TFF3 level was analyzed by using a modified sandwich enzyme‐linked immunosorbent assay. Baseline characteristics, TFF3 level, and salivary flow rate were compared between both groups. Factors associated with the TFF3 level and flow rate were computed by using multivariate linear regression analysis.

**Results:**

Twenty‐eight participants were recruited in the study: 20 patients with OSA (71.42%) and 8 non‐OSA as control. The TFF3 and salivary flow rates between both groups of non‐OSA versus OSA were comparable (TFF3 non‐OSA 61.06 vs. OSA 96.00 ng/mg; *p* = .276 and flow rate non‐OSA 0.40 vs. OSA 0.35 mL/min; *p* = .320). Factors associated with the TFF3 level were neck circumference with a negative coefficient of −16.419 (*p* =  .042). For the salivary flow rate, only age was a significant factor with the coefficient of −0.013 (*p* = .044).

**Conclusions:**

TFF3 and salivary flow rate were comparable between patients with OSA and non‐OSA. The factor associated with TFF3 level was neck circumference, while age was negatively associated with the salivary flow rate in patients with OSA.

## 1 BACKGROUND

Obstructive sleep apnea (OSA) is one of the most common chronic diseases affecting the quality of life of nearly one billion people worldwide (Benjafield et al., 2019). Many recent studies have highlighted an increase in the prevalence of OSA, paralleling the obesity and type 2 diabetes epidemics (Lévy et al., 2015). OSA is now recognized as an independent risk factor for cardiovascular and metabolic diseases but is largely underdiagnosed. OSA is independently associated with the prevalence and incidence of hypertension, stroke, coronary artery disease, heart failure, atrial fibrillation, and metabolic syndrome (Drager et al., 2017; Khamsai, Chootrakool, et al., 2021; Khamsai, Mahawarakorn, et al., 2021; Khamsai, Kachenchart, et al., 2021; Lévy et al., 2015; Sanlung et al., 2020; Soontornrungsun et al., 2020). Because OSA is a multi‐etiologic disorder with a variety of risk factors, symptoms, and treatment options, phenotyping OSA could help in deciding the treatment options ranging from nonsurgical to surgical modalities.

Intraorally, OSA is associated with structural changes in the uvula and soft palate (Paulsen et al., 2002), the influence of the upper airway lining liquid on surface tension is a factor contributing to upper airway collapsibility (Kirkness et al., 1985, 2005; Paulsen et al., 2002; Wang et al., 2014) due to increased airway resistance (Lévy et al., 2015). Previous studies reported trefoil factor family (TFF) peptides: TFF1, TFF2, and TFF3 have lectin activities and are predominantly co‐secreted together with mucins from mucous epithelia (Hoffmann, 2020; Hoffmann et al., 2001). While TFF1 and TFF2 are mainly expressed in the gastric mucosa, TFF3 is rather widely secreted from mucous epithelia and their glands. TFF3 plays an important role in mucous viscosity as well as protective effects such as proliferation and migration enhancement, antiapoptosis, and wound healing, particularly in mucosal surfaces (Hoffmann et al., 2001). Therefore, these mucous epithelia are protected by complex mucous barrier layers, which are part of the innate immune defense (Hoffmann, 2020). TFF3 was also shown to be present in the oral cavity. It is secreted from both major and minor salivary and hence, is a constituent in human saliva (Storesund et al., 2009). Western blot analysis only detected TFF3 in submandibular glands, but not in sublingual and parotid glands (Jagla et al., 1999). Immunofluorescence localized TFF3 uniquely in the secretory granules of serous cells of submandibular glands but not in mucous cells. This localization is remarkably similar to that of the unique low molecular weight mucin MUC5B, which interacts with a number of oral microorganisms (Jagla et al., 1999; Wiede et al., 2001). This suggests a co‐secretion of TFF3 and mucin.

Recent findings conducted in the German population demonstrated a reduction of TFF3 production by the epithelium and subepithelial mucous glands of the uvula. The reduction of TFF3 could contribute to an increase in breathing resistance due to changes in mucous organization, although reduced TFF3 could result in lower mucus viscosity. Moreover, results from enzyme‐linked immunosorbent assay (ELISA) indicated that the TFF3 protein level ranged from 38.1 to 217.8 ng/mg in healthy, snoring, mild/moderate/severe OSA groups. Healthy subjects presented a significantly higher level of TFF3 than snoring and other combined OSA groups. Logistic regression confirmed a correlation between TFF3 concentration and OSA level. Thus, they suggest the involvement of TFF3 in the pathogenesis of OSA (Siber‐Hoogeboom et al., 2017).

Both TFF3 peptides and salivary flow rate are related to salivary glands and may be involved in the pathogenesis of OSA. As there are few studies of both factors and OSA in limited ethnicities, this study aimed to evaluate differences in TFF3 and flow rate between those with and without OSA. Additionally, factors associated with TFF3 and flow rate levels in patients with OSA were also studied.

## 2 METHODS

### 2.1 Subject selection and recruitment

This was a cross‐sectional, experimental study conducted at the Faculty of Medicine and Faculty of Dentistry, Khon Kaen University. The study was approved by the Ethics Committee in Human Research, Khon Kaen University (reference number HE611622). All subjects provided written informed consent before recruitment and their anonymity was maintained throughout the duration of the study. This study was conducted between March and October 2019.

Patients with OSA and non‐OSA were enrolled to the study. OSA patients aged between 25 and 80 years old, diagnosed by the presence of an apnea–hypopnea index (AHI) of five times/h or more by polysomnography, were recruited before starting any treatment modalities from an outpatient Sleep clinic of the university hospital. A non‐OSA group of healthy participants with an AHI of <5 times/h served as a control group. The control group was recruited actively by the study advertisement. Participants with previous OSA treatment, oral lesions, or salivary gland dysfunction were excluded from both the non‐OSA and OSA groups.

Eligible participants were assessed for baseline characteristics, physical signs, and laboratory results, including TFF3, and salivary flow rate. Baseline characteristics include age, gender, OSA risk assessment using the Berlin questionnaire, Epworth Sleepiness Scale (ESS), history of snoring, comorbid conditions, and periodontal condition. History of snoring was self‐reported, while comorbid conditions were collected from medical charts. OSA patients with comorbid diseases were allowed to take regular medication. The Berlin questionnaire was used to classify OSA risk as low and high risk, while ESS indicated a sleepiness score ranging from 0 to 24. Physical signs were blood pressure, body mass index (BMI), and neck/wrist/waist circumferences. The periodontal condition was evaluated by probing depth measurement in all remaining teeth. Pocket depth ≥4 mm indicates periodontitis. An AHI score was also recorded by polysomnography. Those with an AHI of five events/h or more were defined as OSA and categorized as mild, moderate, and severe with the AHI of 5–14, 15–29, and 30 times/h or more, respectively. The unstimulated whole‐saliva samples were collected and subsequently measured salivary flow rate and analyzed for the TFF3 level.

### 2.2 Whole saliva collection and salivary flow rate measurement

Unstimulated whole saliva was collected after participants sat at rest for at least 5 min, rinsed their mouths two times with water, and had not consumed any food or drink for 1 h before saliva collection. Once participants were ready, they were asked to sit and hold a 50 mL centrifuge tube while spitting out whole saliva for 10 min. The salivary flow rate was then measured (mL/min). We measured the unstimulated salivary flow rate by using the technique recommended by Navazesh (1993). The centrifuge tubes were placed in an ice container and immediately transported to the laboratory. The saliva samples were vortexed for 1 min before centrifugation at 10,000*g* under 4°C for 5 min. The supernatant was aliquoted into a 2 mL tube and stored at −80°C until TFF3 concentration (ng/mg) was measured using modified sandwich ELISA (Chaiyarit, Chayasadom, et al., 2012). The total protein concentration in each sample was determined using the Bradford protein assay.

### 2.3 Salivary TFF3 level detection

To quantify salivary TFF3 levels, we used our previously generated monoclonal antibody (mAb) clones 286 and 116 for a modified sandwich ELISA technique (Chaiyarit, Chayasadom, et al., 2012). Briefly, fluorescein isothiocyanate (FITC) and anti‐FITC identification methods with HRP conjugated (1:5000) were used to develop sensitivity. An anti‐human TFF3 mAb clone 286 was used as a plate‐coated antibody to capture TFF3 peptides in saliva samples (50 µg/mL). The antihuman TFF3 mAb clone 116 was used to detect the captured TFF3 peptides. Horseradish peroxidase (HRP)‐conjugated sheep anti‐FITC antibody was used along with TMB substrate to measure color change. The color intensity was measured using a microplate reader at 450 nm. A standard curve was constructed showing salivary TFF3 between 0.5 and 32 ng/mL. All salivary TFF3 concentration data were normalized to total salivary protein concentration and expressed as TFF3 concentration (ng)/salivary protein concentration (mg). %TFF3 in total salivary protein was calculated.

### 2.4 Statistical analyses

Descriptive statistics were used to report baseline characteristics. Shapiro–Wilk test was used to evaluate data normality for numerical factors. Those with normally distributed, data were presented as mean (SD), while those with non‐normally distributed, data were presented as median (range). The inferential statistics were used to calculate differences between those with and without OSA. An independent *t*‐test was used to compare differences between both groups for numerical factors with normal distribution, while the Wilcoxon rank‐sum test was used for numerical variables with non‐normally distributed data. The Fisher Exact test was used to calculate differences between both groups for categorical variables. The TFF3 level and flow rate in patients with and without OSA were compared by using non‐snorer control and OSA with various degrees of OSA severity. Note that the non‐snorer control group was the same control group with the exclusion of two snorers. Comparisons among various OSA severity and control groups were executed by one‐way analysis of variance using the Bonferroni method.

Factors associated with TFF3 level and flow rate in patients with OSA were computed by using multivariate linear regression analysis: stepwise approach. Those with multicollinearity were excluded from the model. Factors with a *p* value of less than 0.20 by a univariate linear regression analysis were entered into the model and factors with a *p* value of less than 0.25 in the multivariate linear regression analysis were remaining in the final model. Results were reported as unadjusted/adjusted coefficients by linear regression analysis with *p* values. All statistical analyses were performed by using STATA software.

## 3 RESULTS

There were 28 participants who were screened, recruited, and met the study criteria: 20 patients with OSA (71.42%) and 8 non‐OSA subjects. The OSA group was significantly different from the non‐OSA group in terms of OSA‐related factors except ESS (Table 1). Co‐morbid diseases were found more in the OSA group (90.00% vs. 50.00%; *p* = .038) than the non‐OSA group. The OSA group also had more proportion of Berlin high‐risk category, snoring, body mass index, neck/wrist/waist circumferences, and AHI than the non‐OSA group. The OSA group had a median AHI of 18.6 events/hour and was categorized as mild (6 patients; 30.00%), moderate (9 patients; 45.00%), and severe (5 patients; 25.00%). Besides, as the OSA group was elder than non‐OSA, 63% of them had periodontal disease with a pocket depth of 4–5 mm or more than 6 mm. The TFF3 and salivary flow rates between both groups were comparable (TFF3 non‐OSA 61.06 vs. OSA 96.00 ng/mg; *p* = .276 and flow rate non‐OSA 0.40 vs. OSA 0.35 mL/min; *p* = .320, respectively). There was no significant difference of TFF3 and flow rate among OSA severities and control groups (Table 2). Note that all groups of OSA severity had higher levels of TFF3 than the control group (mild 105.23; moderate 96.29; severe 84.38 ng/mg of TFF3 vs. 61.06 ng/mg in the control group; *p* =  .276).

**Table 1:** Baseline characteristics, physical signs, laboratory results, trefoil factor family peptide 3 (TFF3), and flow rate of patients with and without obstructive sleep apnea.

Factors	Non‐OSA, *n* = 8	OSA, *n* = 20	*p* Value
Age, years	49.00 (4.87)	57.15 (11.19)	.060
Male gender, *n* (%)	7 (87.50)	11 (55.00)	.194
Berlin high risk, *n* (%)	0	14 (77.78)	.001
ESS	6.88 (3.04)	7.83 (3.95)	.550
Snoring, *n* (%)	2 (40.00)	18 (90.00)	.038
Co‐morbid diseases, *n* (%)	4 (50.00)	18 (90.00)	.038
Allergic rhinitis, *n* (%)	1 (12.50)	0	.286
Stroke, *n* (%)	0	1 (5.26)	.999
Periodontal status, *n* (%)			.663
No pockets	4 (50.00)	7 (36.84)	
Pockets 4–5 mm	3 (37.50)	6 (31.58)	
Pockets ≥6 mm	1 (12.50)	6 (31.58)	
Systolic blood pressure, mmHg	111.38 (16.77)	134.90 (18.24)	.004
Diastolic blood pressure, mmHg	65.00 (11.38)	73.50 (12.36)	.105
BMI, kg/m^2^	21.18 (20.00–24.50)	25.80 (18.21–48.83)	.001
Neck circumference, cm	32.44 (2.64)	38.35 (4.61)	.002
Wrist circumference, cm	15.38 (1.16)	17.00 (1.73)	.022
Waist circumference, cm	75.00(5.53)	97.28 (15.33)	<.001
AHI, times/h	2.2 (1.2–3.5)	18.6 (5.4–81.0)	.001
TFF3, ng/mg	61.06 (48.41)	96.00 (82.68)	.276
Flow rate, mL/min	0.40 (0.23–0.95)	0.35 (0.13–1.51)	.320

*Note*: Data presented as median (range) or mean (SD) unless indicated otherwise.

Abbreviations: AHI, apnea–hypopnea index; BMI, body mass index; ESS, Epworth Sleepiness Scale.

**Table 2:** Trefoil factor family peptide 3 (TFF3) level and salivary flow rate in patients with and without obstructive sleep apnea (OSA).

Factors	Non‐OSA	OSA, *n* = 20	*p* Value
TFF3, ng/mg^a^	*n* = 8	*n* = 20	
	61.06 (48.41)	96.00 (82.68)	.276
		105.23 (99.55), *n* = 6^b^	.638^c^
		96.29 (74.25), *n* = 9^d^	
		84.38 (93.57), *n* = 5^e^	
	*n* = 6 (non‐snorers)	*n* = 20	
	68.01 (50.82)	96.00 (82.68)	.443
		105.23 (99.55), *n* = 6^b^	.861^c^
		96.29 (74.25), *n* = 9^d^	
		84.38 (93.57), *n* = 5^e^	
Flow rate, mL/min^f^	*n* = 8	*n* = 20	
	0.40 (0.23–0.95)	0.35 (0.13–1.51), *n* = 20	.320
		0.35 (0.13–0.43), *n* = 6^b^	.233^c^
		0.42 (0.18–1.51), *n* = 9^d^	
		0.30 (0.18–0.54), *n* = 5^e^	
	*n* = 6 (non‐snorers)	*n* = 20	
	0.62 (0.35–0.95)	0.34 (0.13–1.51)	.058
		0.35 (0.13–0.43), *n* = 6^b^	.147^c^
		0.42 (0.18–1.51), *n* = 9^d^	
		0.30 (0.18–0.54), *n* = 5^e^	

*Note*: The non‐OSA group had two groups: all control (*n* = 8) and non‐snorers (*n* = 6).

^a^ Data presented as mean (SD).

^b^ Mild OSA.

^c^ one‐way analysis of variance with no significant pairwise comparison by Bonferroni method.

^d^ Moderate OSA.

^e^ Severe OSA.

^f^ Data presented as median (range).

Multivariate linear regression analysis showed that there were two factors remaining to be associated with TFF3 level: age and neck circumference (Table 3). Only neck circumference was independently associated with TFF3 level with a negative coefficient of −16.419 (*p* = .042). For flow rate, there were three factors remaining in the final model (Table 4). But, only age was a significant factor with the coefficient of −0.013 (*p* = .044).

**Table 3:** Factors associated with trefoil factor family peptide 3 (TFF3) level and salivary flow rate in patients with obstructive sleep apnea (OSA) by linear regression analysis.

Factors	Unadjusted coefficient (95% confidence interval; *p* value)	Adjusted coefficient (95% confidence interval; *p* value)
Age	−2.788 (−6.178, 0.600; .101)	−6.791 (−15.809, 2.227; .105)
Neck circumference	−6.645 (−14.891, 1.600; .108)	−16.419 (−31.882, −0.956; .042)

**Table 4:** Factors associated with a salivary flow rate in patients with obstructive sleep apnea (OSA) by linear regression analysis.

Factors	Unadjusted coefficient (95% confidence interval; *p* value)	Adjusted coefficient (95% confidence interval; *p* value)
Age	−0.014 (−0.026, −0.001; .033)	−0.013 (−0.026, −0.001; .044)
Neck circumference	0.019 (−0.014, 0.052; .240)	0.027 (−0.011, 0.064; .149)
Apnea–hypopnea index	−0.002 (−0.011, 0.007; .711)	−0.008 (−0.018, 0.002; .101)

## 4 DISCUSSION

Previous studies suggested that hypoxia is associated with an increased TFF3 mRNA expression in intestinal epithelial cells as a mechanism to maintain a barrier function when oxygen levels are low (Furuta et al., 2001; Louis et al., 2006). OSA patients commonly encounter chronic hypoxia resulting in lower oxygen saturation. Therefore, the production of TFF3 mRNA in oral epithelial cells may as well be increased. However, our study demonstrated that there was no difference in TFF3 level between those with and without OSA regardless of OSA severity (Table 2).

The nonsignificant increasing TFF3 level in this study is contrary to the previous study of TFF3 in patients with snoring and OSA by Siber‐Hoogeboom et al. (2017) in Germany who reported a reduced TFF3 level. This could be owing to the fact that being OSA with chronic hypoxia may increase TFF3 mRNA expression and TFF3 level to maintain a barrier in oral epithelial cells. However, this increasing level was not large enough to be different from the non‐OSA group. We tested for the correlation of TFF3 and oxygenation data from polysomnography to prove this association by Pearson correlation (Table 5). However, the TFF3 level was not significantly related to any oxygenation data.

**Table 5:** Pearson correlation between trefoil factor family peptide 3 (TFF3) level and polysomnographic factors.

Factors	Pearson correlation coefficients	*p* value
Apnea–hypopnea index, times/h	−0.012	.950
Desaturation index, times/h	−0.335	.287
Lowest oxygenation, %	0.1096	.721
Average oxygenation, %	0.373	.189
Time with oxygenation <90%, %	−0.357	.255

TFF3 is a biomarker for disease activity in patients with ulcerative colitis (Grønbæk et al., 2006; Nakov, Velikova, Nakov, Gerova, et al., 2019; Nakov, Velikova, Nakov, Ianiro, et al., 2019). The TFF3 level was significantly higher in active ulcerative colitis compared with quiescent ulcerative colitis (10.12 vs. 6.48 ng/mL; *p* < .001) (Nakov, Velikova, Nakov, Ianiro, et al., 2019). These findings may indicate that TFF3 is upregulated in the inflammatory mucous epithelium of the gastrointestinal tract and oral mucosa. A previous study found that patients with mild to moderate OSA and severe OSA had significantly higher proportions of plaques versus the control group (59% and 73% vs. 38%; *p* = .001) (Tranfić Duplančić et al., 2022). Dental plaques, containing over 700 bacteria, lead to local inflammation resulting in higher TFF3 levels in patients with OSA (Gao et al., 2018; Murakami et al., 2018; Paul et al., 2021). The results of this study were opposite from the previous study (Siber‐Hoogeboom et al., 2017). These differences may be due to the different methods being used in both studies. We used the in‐house ELISA methods, while the previous study used the commercial kit from Cloud‐Clone Corp (Wuhan, China). Our methods were similar to previous studies, (Chaiyarit, Chayasadom, et al., 2012; Chaiyarit, Utrawichian, et al., 2012) while the quality of the commercial kit was not reported in the previous article. As both studies had small sample sizes, further studies with larger sample sizes for both methods are required.

Our results also showed that neck circumference appeared to be negatively associated with TFF3 level, respectively (Table 2). The previous study found that severe OSA had associated with a reduction of TFF3 level (Siber‐Hoogeboom et al., 2017). Our study found that a larger neck circumference was associated with lower TFF3 levels (Table 2). These findings may explain by the correlation between neck circumference and the severity of OSA. A previous study demonstrated that neck circumference is a predictor of OSA severity: mild (37.1 cm), moderate (38.0 cm), and severe (39.8 cm) with a *p* value of 0.0001 (Oliveros et al., 2021). The previous study found that severe OSA was associated with a reduction of TFF3 levels (Siber‐Hoogeboom et al., 2017). In contrast, we found that being OSA was associated with increasing of TFF3. These findings may be due to the difference in the study population. This study had average AHI of only 18.6 times/h which was not classified as severe OSA. Additionally, OSA in the Asian population had a proportion of obesity of approximately 50% (Chirakalwasan et al., 2013). As seen in this study, the average body mass index was only 25.80 kg/m^2^. These two factors may cause different effects of OSA on TFF3 in both studies. These findings may also indicate that it might be other mechanisms in the correlation of OSA and TFF3 levels. The results of this study were similar to other previous studies in that OSA may have increasing TFF3 as high TFF3 indicating of higher mucus viscosity in mucosal epithelia and saliva leading to airway collapse in patients with OSA (Bastholm et al., 2017; Hoffmann, 2021; Verey et al., 2011).

Regarding flow rate, there was no significant difference between the OSA and non‐OSA groups but slightly lower in the OSA group (0.35 vs. 0.40 mL/min) as shown in Table 1. Our analysis also found that age was negatively associated with the flow rate. The decrease in salivary flow rate could be explained by aging‐related changes in quantity (flow rate) and quality (e.g., ion and protein composition, rheology, tribology) of saliva (Xu et al., 2019). As the average age in OSA group was older than the non‐OSA group in our study (57 vs 49 years), the salivary flow rate was lower in the OSA group than the non‐OSA group. The decrease in salivary flow supports by a recent observational study in 30 OSA patients aged 35–65 years reported that most of the patients complained about dry mouth (73.3%) and there was hyposalivation detected in 20% of them. Dry mouth on awakening was observed in 60.0%, 72.7%, and 88.9% of patients with mild, moderate, and severe OSA, respectively. The average salivary flow rate was 0.28, 0.24, and 0.14 mL/min, respectively. The average pH value in patients with mild, moderate, and severe apnea was 6.40 ± 0.017, 6.15 ± 0.27, and 5.87 ± 0.24, respectively. The acidity of the saliva was correlated with the level of OSA, and it is statistically increased with the increment of the OSA severity (Makeeva et al., 2021).

The strength of our study was enhanced by the multivariate model analyses. However, there are some limitations in this study. First, the sample size was small indicating preliminary results. However, significant predictors for TFF3 and salivary flow rate were present. Factors related to OSA and its treatment were not evaluated. Further larger studies are required to confirm the results of this study as this study population was collected from the university hospital setting. The results may not be generalized for the general population. Finally, there was no sample size calculation. With respect to the nonsignificant results, the study may not have enough statistical power to test its hypothesis.

## 5 CONCLUSIONS

TFF3 and salivary flow rate were comparable between patients with OSA and non‐OSA controls. The factor associated with TFF3 level was neck circumference, while age was negatively associated with the salivary flow rate in patients with OSA.

## AUTHOR CONTRIBUTIONS

*Conception and design of study*: Supanigar Ruangsri. *Acquisition of data*: Supanigar Ruangsri, Gintawat Doolgindachbaporn, Worrapon Chokwatwikul, and Kanchanaporn Wattanawareekul. *Analysis and/or interpretation of data*: Supanigar Ruangsri, Gintawat Doolgindachbaporn, Worrapon Chokwatwikul, Kanchanaporn Wattanawareekul, Subin Puasiri, and Kittisak Sawanyawisuth. *Drafting the manuscript*: Supanigar Ruangsri. *Revising the manuscript critically for important intellectual content*: Kittisak Sawanyawisuth. *Approval of the version of the manuscript to be published*: Supanigar Ruangsri, Gintawat Doolgindachbaporn, Worrapon Chokwatwikul, Kanchanaporn Wattanawareekul, Subin Puasiri, and Kittisak Sawanyawisuth.

## CONFLICT OF INTEREST STATEMENT

The authors declare no conflict of interest.

## Data Availability

Data that support the findings of this study are available from the corresponding author, Kittisak Sawanyawisuth, upon reasonable request.
